# Supplementary material for: Moisture-enabled self-charging and voltage stabilizing supercapacitor
Source: Nat Commun. 2024 Jun 10;15:4929. doi: 10.1038/s41467-024-49393-9 (PMC11165001; doi:10.1038/s41467-024-49393-9)
Supplement: Supplementary file 1 — Supporting Information [file 41467_2024_49393_MOESM1_ESM.pdf]

Supplementary Information

Moisture-enabled self-charging and voltage stabilizing supercapacitor

Lifeng Wang[1,2,3,4], Haiyan Wang[2], Chunxiao Wu[1,2], Jiaxin Bai[4], Tiancheng He[4], Yan Li[1,*], Huhu Cheng[2, 4, 5*], Liangti Qu[2, 4, 5*]

[1]School of Materials Science and Engineering, University of Science and Technology Beijing, Beijing, P.R. China

[2]Key Laboratory of Organic Optoelectronics & Molecular Engineering, Ministry of Education, Department of Chemistry, Tsinghua University, Beijing 100084, China.

[3]State Key Laboratory of Transient Optics and Photonics, Xi'an Institute of Optics and Precision Mechanics, Chinese Academy of Sciences, Xi'an, P. R. China

[4]State Key Laboratory of Tribology in Advanced Equipment (SKLT), Department of Mechanical Engineering, Tsinghua University, Beijing 100084, China.

[5]Laboratory of Flexible Electronics Technology, Tsinghua University, Beijing 100084, P. R. China.

Email: Yan Li (liyan2011@ustb.edu.cn), Huhu Cheng (huhucheng@tsinghua.edu.cn), Liangti Qu (lqu@mail.tsinghua.edu.cn)

Theoretical modeling.

To understand the relationship with ions diffusion process and the electric power generation performance, the COMSOL simulations are performed for the polyelectrolyte film. A 1D model with the height of 100 μm (PDDA: 30 μm, PSS 70 μm) was set up based on the experiment parameters. 'Electrostatics' and the 'Transport of diluted species' modules were adopted governing by the Nernst-Planck-Poisson equations:

$$j_i = D(\nabla_{c_i} + \frac{z_i F c_i}{RT} \nabla \varphi) \quad (1)$$

$$\nabla^2 \varphi = -\frac{F}{\varepsilon} \sum z_i c_i \quad (2)$$

$$\nabla \cdot j_i = 0 \quad (3)$$

where, j_i, D, c_i, z_i, φ, and ε are the ionic flux, diffusion coefficient, ion concentration, valence number for each species i, electrical potential, and dielectric constant of the polyelectrolyte film, respectively. The parameters were set based on the experiment tests or the previous reports.[1-3] The ion concentration of H^+ and Cl^- for PSS and PDDA were assumed as 5×10^{-6} mol m^{-3} and 1×10^{-6} mol m^{-3}, respectively. The diffusion coefficient for H^+ and Cl^- for were presumed to be 5×10^{-11} m^2 s^{-1}.

Supplementary Figures

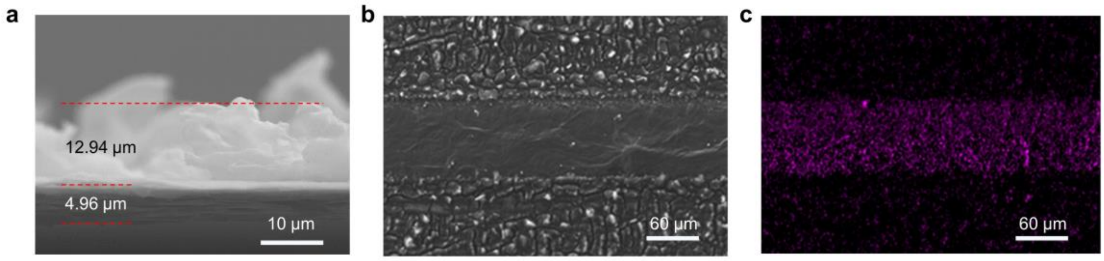

Supplementary Fig. 1 Characterization of the laser reduced rGO. The scanning electron microscopy (SEM) images of the cross-section (**a**) and the surface (**b**) of the rGO electrodes and the corresponding energy-dispersive X-ray spectroscopy (EDS) mapping of the O element (**c**). After laser treatment, GO was rapidly reduced to a porous rGO structure due to the thermal effect of the laser [4, 5], which adhered to the GO film closely. The thickness of the rGO is ~12.94μm. Compared to the dense and smooth GO film, the porous structure of rGO can increase the specific surface area of the active material and provide more ion adsorption sites, while the porous structure also benefits the infiltration of the electrolyte and ion transport. EDS mapping of the O element showed that laser direct writing significantly reduced the oxygen content in GO.

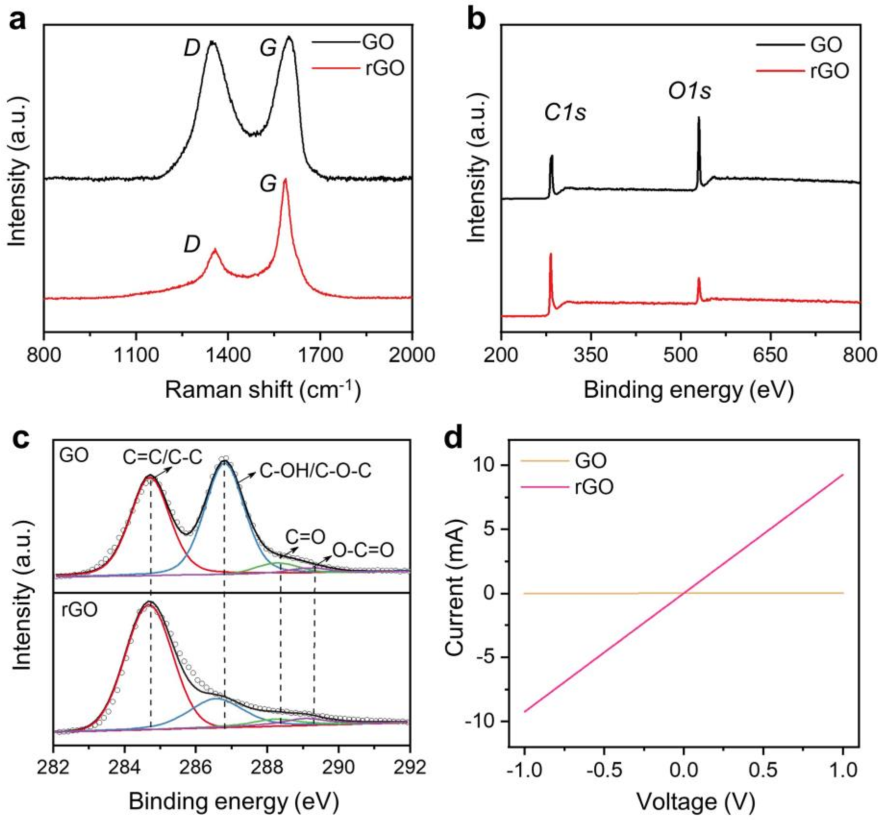

Supplementary Fig. 2 Chemical structural changes of rGO microelectrodes obtained by laser direct writing. Raman spectra (**a**), X-ray photoelectron spectroscopy (XPS) spectra (**b**), C 1s narrow spectra (**c**), and I-V curves (**d**) of GO and rGO obtained by laser reduction. Raman spectroscopy demonstrated that after laser reduction, the I_D/I_G of rGO was 0.38, significantly lower than that of the GO membrane (0.97), indicating a substantial decrease in the defect content. XPS analysis revealed that the proportion of O atoms in rGO microelectrodes decreased from 31.29% (GO membrane) to 14.79%. The O 1s narrow spectrum in Fig. S2c confirmed that the reduction in oxygen content was mainly due to the decrease in C-OH/C-O-C functional groups, which decreased by 55.2%. The significant decrease in the proportion of oxygen functional groups and the reduction in defect level increased the electronic conductivity (from 5.09×10^{-3} S m^{-1} to

2725.56 S m^{-1}). The highly conductive and porous rGO can serve as both an active material for EC to provide capacity and as a current collector.

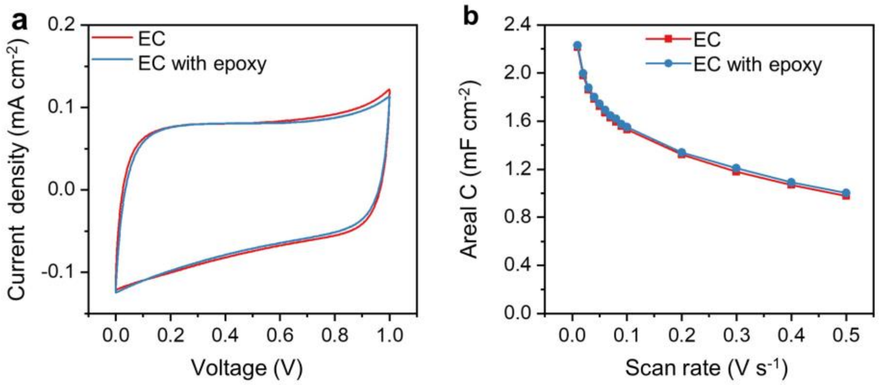

Supplementary Fig. 3 The electrochemical performance of the initial EC and the EC coated by epoxy. CV curves (**a**) and the corresponding areal capacitance of the initial EC and the EC coated by epoxy (**b**). As shown in Fig. S3a and S3b, the epoxy coating layer barely affects the electrochemical performance of the EC.

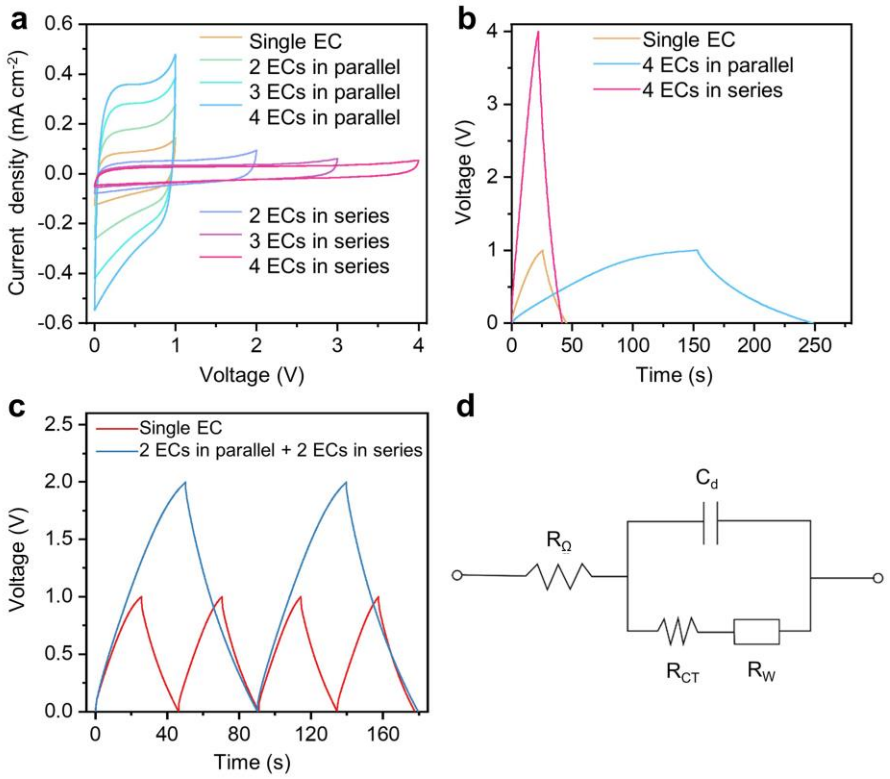

Supplementary Fig. 4 The integrated performance of the EC. The CV profiles (**a**) and galvanostatic charge/discharge curves (**b**) of four ECs connected in series or parallel. **c** The galvanostatic charge/discharge curves of four ECs connected in a combination of series and parallel. **d** The equivalent circuit of the EC.

To meet the practical application requirements, ECs can increase the output voltage by connecting them in series or increase the capacity by connecting them in parallel. As shown in Supplementary Fig. 4a and b, by connecting 4 ECs in series, the voltage window can reach 4 V. After connecting 4 ECs in series, the discharge time is extended by about 4 times compared to a single device. Supplementary Fig. 4c shows four EC connected in a mixed combination of series and parallel to achieve higher power density and energy density. The voltage and discharge time doubled with the combined two ECs in series and two ECs in parallel. The equivalent series resistance

of the supercapacitor (Supplementary Fig. 4d) is composed of three parts, including intrinsic ohmic resistance (R_Ω, 302 Ω cm^2), interfacial charge transfer resistance (R_{CT}, 24 Ω cm^2) and Warburg diffusion resistance (R_W, 160 Ω cm^2).

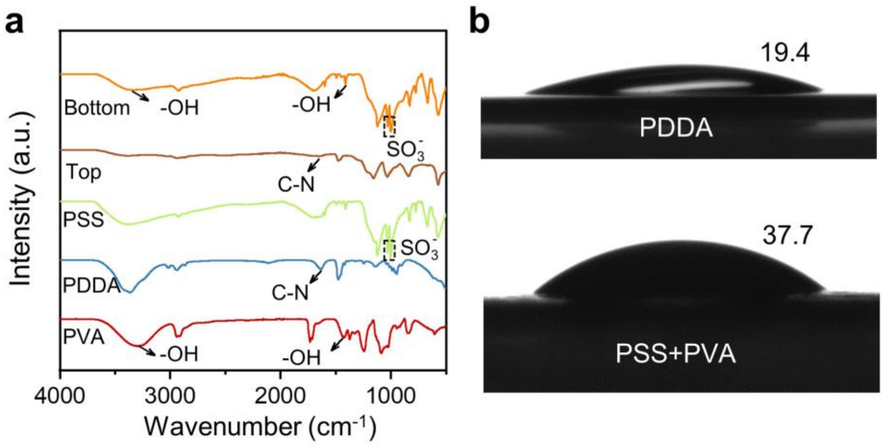

Supplementary Fig. 5 Characterization of the bilayer polyelectrolyte film. **a** Fourier transform infrared spectra (FT-IR) of the top and bottom surfaces of the film. **b** Contact angle photos of PDDA and PSS+PVA membranes.

The FT-IR analysis reveals the characteristic absorption peaks of -OH for PVA at 1420 cm^{-1} and 3260 cm^{-1},[6] C-N for PDDA at 1635 cm^{-1}, [7] and $-SO_{3-}$ for PSS at 1002 cm^{-1} and 1030 cm^{-1}.[8] Moreover, the C-N stretching vibration peak of PDDA was detected on the upper surface of the bilayer film, while the -OH and $-SO_{3-}$ stretching vibration peaks of PVA and PSS were detected on the lower surface, respectively. These features confirm the successful preparation of the bilayer heterogeneous membrane structure. The abundant hydrophilic functional groups endow the polyelectrolyte membrane with excellent hydrophilicity and water adsorption ability. The contact angle of the PDDA film at the top of the polyelectrolyte membrane is 19.4°,

while the contact angle of the PSS+PVA mixed film at the bottom is 37.7°, indicating excellent hydrophilicity of the polyelectrolyte film.

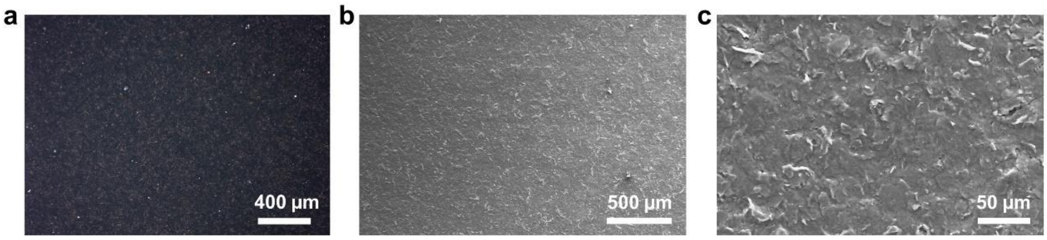

Supplementary Fig. 6 The surface morphology of the carbon electrode. **a** The optic photo of the blade coating carbon electrode. SEM images of the carbon electrode (**b-c**).

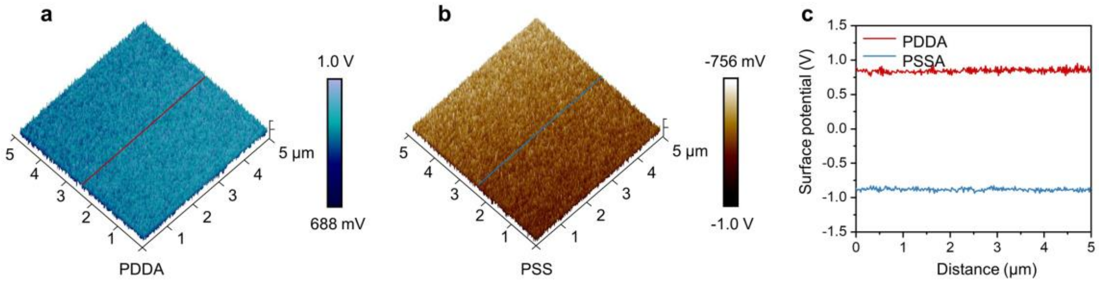

Supplementary Fig. 7 Alterations in surface potential of PDDA and PSS following water absorption. Kelvin probe force microscopy images of PDDA (**a**) and PSS (**b**) after absorbing water, respectively. **c** The surface potential of PDDA and PSS film.

Supplementary Fig. 7a and b show the surface potential of PDDA and PSS after water absorption. It can be seen that the polymer backbone left behind after the migration of migratable chloride or hydrogen ions produces a surface potential of 0.92 V and -0.87 V for PDDA and PSS (Supplementary Fig. 7c), respectively. The surface potential difference corroborates the electromotive force produced as a result of the concentration gradient of ions.

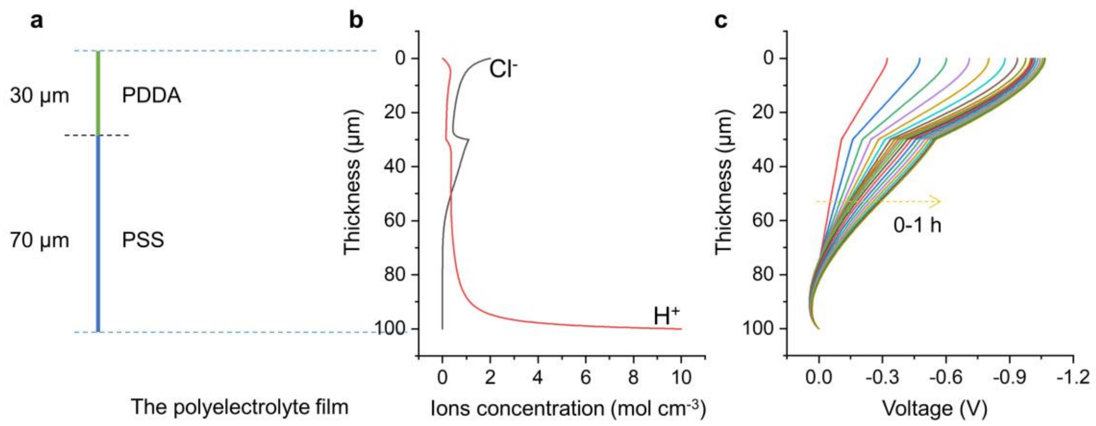

Supplementary Fig. 8 The simulation results of ion diffusion and electric power generation. **a** Numerical simulated model of the polyelectrolyte film. **b** The H^+ and Cl^- ions distribution in the polyelectrolyte film. **c** The electric power generation process with the ions diffusion.

After absorbing water, the polymeric electrolytes can gradually undergo dissociation. The long-chain structure of the polymer backbone is difficult to migrate, while the dissociated anions and cations from the polycation and polyanion are migratable and could spontaneously diffuse due to the concentration gradient, resulting in a potential difference within the polyelectrolyte membrane. The numerical simulation confirms the asymmetrical distribution of H^+ and Cl^- ions in the polyelectrolyte film. As ions gradually diffused, a potential difference of 1.07 V was established across the terminals of the polyelectrolyte film after a duration of one hour, consistent with the experimental results.

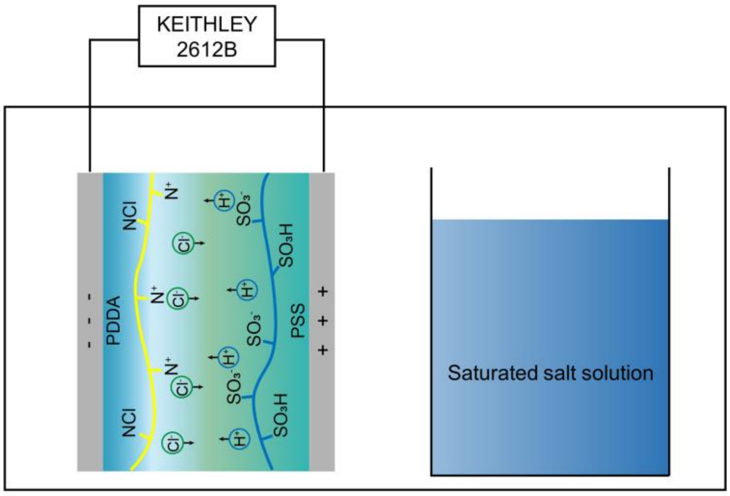

Supplementary Fig. 9 Experimental setup for the MEG. Saturated salt solutions were used to regulate the relative humidity in the testing system.

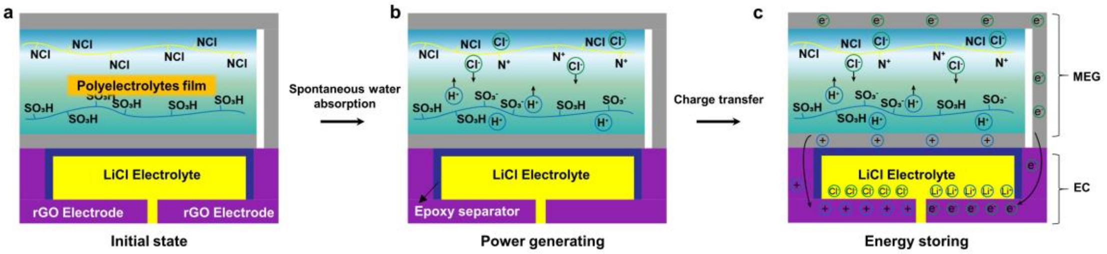

Supplementary Fig. 10 Schematic of the charging process of the mp-SC. **a** The initial state of the mp-SC. **b** The spontaneous water absorption and ion transport processes. **c** The ions in electrolyte within the EC selectively adsorbs onto the surface of the rGO electrodes under the applied voltage bias.

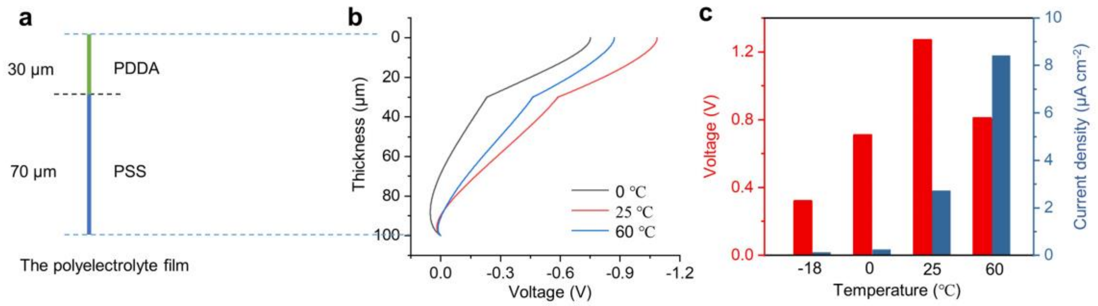

Supplementary Fig. 11 The power generation performance of the polyelectrolyte-based MEG. **a** Numerical simulated model of the polyelectrolyte film. **b** The simulation results depict how the distribution of the electric field within the polyelectrolyte film varies with changes in temperature. **c** The output voltage and current densities of the the polyelectrolyte moist-electric generator at different temperatures.

Supplementary Table 1 Comparison of the power density of the mp-SC and the recently reported moisture power generators.

Materials	Device structures	Electrodes	Voltage (V)	Power density (μW cm^{-2})	Reference
GO film-MEG	Thin film	Ag	0.7	27	9
PSS-MEG	Thin film	steel@Au	0.8	17	2
GON-MEG	Thin film	Al	0.04	12	10
PSS+PVA-MEG	Thin film	Ag NWs	0.6	7.9	8
PSS/PVA-MEG	Thin film	carbon tape	0.95	5.5	3
TiO_2-MEG	Nanowire networks	Ag NW	0.50	4	11
PPy-MEG	Foam	Au	0.06	0.69	12
g-GOF-MEG	Film	Au	0.04	0.42	13
IPMEG-MEG	Film	rGO	0.18	0.1	4
GO/PAAS-MEG	Bulk	Au/Ag	0.60	0.07	1
a-GOM-MEG	Foam	Au	0.45	0.0184	14
Cellulose-MEG	Nanofibrous foam	Pt	0.11	3E-4	15
mp-SC	Thin film	Carbon	0.9	49.38	This work

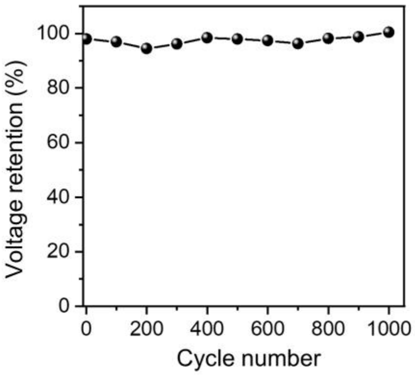

Supplementary Fig. 12 The voltage retention rate of the mp-SC after cyclic bending test.

The mp-SC exhibited excellent flexibility. After bending the device 180°, the voltage remained almost unchanged and the stability was even higher than that of the MEG. Moreover, after 1000 cycles of bending, the voltage retention rate of the device was close to 100%, demonstrating its excellent flexibility and voltage stability.

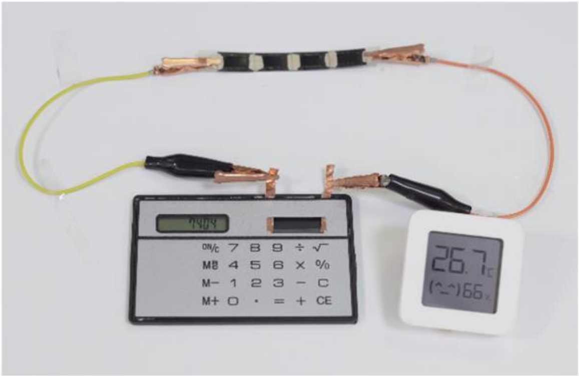

Supplementary Fig. 13 Commercial electronic calculator powered by four mp-SCs connected in serial after self-charging in the ambient environment.

Supplementary References

1. Huang Y, Cheng H, Yang C, Yao H, Li C, Qu L. All-region-applicable, continuous power supply of graphene oxide composite. *Energ. Environ. Sci.* **12**, 1848-1856 (2019).
2. Xu T*, et al.* An efficient polymer moist-electric generator. *Energ. Environ. Sci.* **12**, 972-978 (2019).
3. Wang H*, et al.* Bilayer of polyelectrolyte films for spontaneous power generation in air up to an integrated 1,000 V output. *Nat. Nanotechnol.* **16**, 811-819 (2021).
4. Yang C, Huang Y, Cheng H, Jiang L, Qu L. Rollable, stretchable, and reconfigurable graphene hygroelectric generators. *Adv. Mater.* **31**, 1805705 (2019).
5. El-Kady MF, Kaner RB. Scalable fabrication of high-power graphene micro-supercapacitors for flexible and on-chip energy storage. *Nat. Commun.* **4**, 1475 (2013).
6. Samsudin AM, Hacker V. Preparation and characterization of PVA/PDDA/nano-zirconia composite anion exchange membranes for fuel cells. *Polymers* **11**, 1399 (2019).
7. Zhang J, Zhou T, Qiao J, Liu Y, Zhang J. Hydroxyl anion conducting membranes poly (vinyl alcohol)/poly (diallyldimethylammonium chloride) for alkaline fuel cell applications: Effect of molecular weight. *Electrochim. Acta* **111**, 351-358 (2013).

8. Wang H*, et al.* Transparent, self-healing, arbitrary tailorable moist-electric film generator. *Nano Energy* **67**, 104238 (2020).

9. Liang Y*, et al.* Electric power generation via asymmetric moisturizing of graphene oxide for flexible, printable and portable electronics. *Energy Environ. Sci.* **11**, 1730-1735 (2018).

10. Zhao F, Wang L, Zhao Y, Qu L, Dai L. Graphene oxide nanoribbon assembly toward moisture-powered information storage. *Adv. Mater.* **29**, 1604972 (2017).

11. Shen D, Xiao M, Zou G, Liu L, Duley WW, Zhou YN. Self-powered wearable electronics based on moisture enabled electricity generation. *Adv. Mater.* **30**, 1705925 (2018).

12. Xue JL*, et al.* Vapor-activated power generation on conductive polymer. *Adv. Funct. Mater.* **26**, 8784-8792 (2016).

13. Zhao F, Cheng H, Zhang Z, Jiang L, Qu L. Direct power generation from a graphene oxide film under moisture. *Adv. Mater.* **27**, 4351-4357 (2015).

14. Cheng HH*, et al.* Spontaneous power source in ambient air of a well-directionally reduced graphene oxide bulk. *Energy Environ. Sci.* **11**, 2839-2845 (2018).

15. Li M*, et al.* Biological nanofibrous generator for electricity harvest from moist air flow. *Adv. Funct. Mater.* **29**, 1901798 (2019).
