# Supplementary material for: Moisture-enabled self-charging and voltage stabilizing supercapacitor
Source: Nat Commun. 2024 Jun 10;15:4929. doi: 10.1038/s41467-024-49393-9 (PMC11165001; doi:10.1038/s41467-024-49393-9)
Supplement: Supplementary file 3 — Description of Additional Supplementary Information [file 41467_2024_49393_MOESM3_ESM.docx]

**Description of Additional Supplementary Files**

File Name: Supplementary Movie 1

Description: The electricity generation performance of the moist-electric generator under 5% RH or 90% humidity conditions.

File Name: Supplementary Movie 2

Description: The electricity generation performance of the mp-Sc under 5% RH or 95% humidity conditions

File Name: Supplementary Movie 3

Description: A wristwatch is driven by three mp-SCs connected in series.

File Name: Supplementary Movie 4

Description: Demonstration of four mp-SCs connected in series as a power-supplying source for directly driving a commercial electronic calculator.

File Name: Supplementary Source Data 1

Description: The raw data generated in this study.
